# FOXP3 Is a HCC suppressor gene and Acts through regulating the TGF-β/Smad2/3 signaling pathway

**DOI:** 10.1186/s12885-017-3633-6

**Published:** 2017-09-13

**Authors:** Jie-Yi Shi, Li-Jie Ma, Ji-Wei Zhang, Meng Duan, Zhen-Bin Ding, Liu-Xiao Yang, Ya Cao, Jian Zhou, Jia Fan, Xiaoming Zhang, Ying-Jun Zhao, Xiao-Ying Wang, Qiang Gao

**Affiliations:** 1 Liver Cancer Institute, Zhongshan Hospital, and Key Laboratory of Carcinogenesis and Cancer Invasion (Ministry of Education), Fudan University, 180 Fenglin Road, Shanghai, 200032 People’s Republic of China; 2 Cancer Research Institute, Fudan University Shanghai Cancer Center, Shanghai, People’s Republic of China; 3 Cancer Research Institute, Xiangya School of Medicine, Central South University, Changsha, Hunan People’s Republic of China; 4 Institute of Biomedical Sciences, Fudan University, Shanghai, People’s Republic of China; 5 Key Laboratory of Molecular Virology & Immunology, Institut Pasteur of Shanghai, Chinese Academy of Sciences, Shanghai, People’s Republic of China

**Keywords:** FOXP3, Hepatocellular carcinoma, TGF-β, Prognosis, ChIP

## Abstract

**Background:**

FOXP3 has been discovered to be expressed in tumor cells and participate in the regulation of tumor behavior. Herein, we investigated the clinical relevance and biological significance of FOXP3 expression in human hepatocellular carcinoma (HCC).

**Methods:**

Expression profile of FOXP3 was analyzed using real-time RT-PCR, western blotting and immunofluorescence on HCC cell lines, and immunostaing of a tissue microarray containing of 240 primary HCC samples. The potential regulatory roles of FOXP3 were dissected by an integrated approach, combining biochemical assays, analysis of patient survival, genetic manipulation of HCC cell lines, mouse xenograft tumor models and chromatin immunoprecipitation (ChIP) sequencing.

**Results:**

FOXP3 was constitutively expressed in HCC cells with the existence of splice variants (especially exon 3 and 4 deleted, Δ3,4-FOXP3). High expression of FOXP3 significantly correlated with low serum α-fetoprotein (AFP) level, absence of vascular invasion and early TNM stage. Survival analyses revealed that increased FOXP3 expression was significantly associated with better survival and reduced recurrence, and served as an independent prognosticator for HCC patients. Furthermore, FOXP3 could potently suppress the proliferation and invasion of HCC cells in vitro and reduce tumor growth in vivo. However, Δ3,4-FOXP3 showed a significant reduction in the tumor-inhibiting effect. The inhibition of FOXP3 on HCC aggressiveness was acted probably by enhancing the TGF-β/Smad2/3 signaling pathway.

**Conclusion:**

Our findings suggest that FOXP3 suppresses tumor progression in HCC via TGF-β/Smad2/3 signaling pathway, highlighting the role of FOXP3 as a prognostic factor and novel target for an optimal therapy against this fatal malignancy.

**Electronic supplementary material:**

The online version of this article (10.1186/s12885-017-3633-6) contains supplementary material, which is available to authorized users.

## Background

FOXP3 is a member of the forkhead family of transcription factors, and initially thought to be restricted to hematopoietic tissues and act as a “master switch” for the development and function of regulatory T Cells (Treg) [1]. Genome-wide analysis of FOXP3^+^ T cells indicated that FOXP3 can bind to and up- or down-regulate a large number of genes and microRNAs, indicating a dual role of FOXP3 as both transcriptional activator and repressor [2, 3]. Recently, reports have demonstrated that FOXP3 is also expressed in tumor cells, suggesting that FOXP3 may have a broader role in cancer than initially thought [4, 5]. However, the biological function and clinical relevance of FOXP3 in tumor cells remain controversial. Data have suggested that FOXP3 expression in tumor cells could be a poor prognostic factor in breast cancer [6], colorectal cancer [4], and bladder cancer [7], indicating FOXP3 significantly contributed to tumor progression. Functional experiments have indicated that melanoma cells could have FOXP3-dependent suppressive effects on T cells [8], and FOXP3 play an important role in progression of cervical cancer cells [9], thus suggesting that FOXP3 expression in cancer cells might trigger a mechanism of immune evasion and tumor progression. In contrast with these data, FOXP3 was demonstrated to be a transcriptional repressor of two breast cancer oncogenes, SKP2 and HER2, acting as a potential tumor suppressor gene [10, 11]. Similar results were reported in prostate cancer [12] and gastric cancer [13]. Thus, it seems that the exact function of FOXP3 acts in a cancer type-specific manner.

Hepatocellular carcinoma (HCC), epidemic to Asia and Africa with an increasing incidence in western countries, is one of the most common and aggressive cancers worldwide [14]. Previously, we and others have demonstrated that high-density of FOXP3^+^ Treg infiltration was associated with tumor aggressiveness and poor clinical outcome in HCC [15, 16]. In our precious studies [15], immunohistochemical staining for FOXP3^+^ Treg in human HCC tissue also revealed positive FOXP3 staining in HCC cells. Thus, an interesting issue is whether FOXP3 gene is a friend or a foe for HCC cells. Interestingly, investigation of heterozygous Scurfy mice Foxp3^sf/+^ revealed that they also harbored hepatoma, in addition to mammary tumors, suggesting that FOXP3 may have an impeditive role in hepatocarcinogenesis [10]. However, the expression and the molecular biological functions of FOXP3 have not been investigated deeply.

In this study, we showed that FOXP3 was consistently expressed in HCC cell lines and was further identified as an independent predictor for better prognosis in HCC patients. FOXP3 reduced tumor growth and invasion in vitro and in vivo, however, the splice variant of FOXP3 led to impaired protective function. In addition, the tumor inhibition of FOXP3 might be regulated by TGF-β signaling pathway via Smad2/3.

## Methods

### Cell lines and transfection

The human HCC cell lines MHCC97H (97H) and MHCC97L (97 L) were established in our laboratory [17, 18]. Huh7 (JCRB0403) was obtained from the Japanese Cancer Research Bank; PLC/PRF/5 (PLC, CRL-8024), HepG2 (HB-8065) and Hep3B (HB-8064) were purchased from the American Type Culture Collection; SMMC-7721 (7721, TCHu52), and human hepatocytes HL-7702 (L02, GNHu 6) were obtained from Cell Bank (Shanghai, China).

Full-length FOXP3 and Δ3,4-FOXP3 cDNA were amplified by PCR from cDNA of 97H cells. Then, the FOXP3 constructs were subcloned into pWPXL vector (Addgene). Lentiviral stocks were prepared by co-transfecting HEK-293 T cells with FOXP3 expression constructs and the corresponding empty vectors, and standard virus packaging systems [19]. Knock-down of FOXP3 was accomplished using shRNA (5′-GCA CAT TCC CAG AGT TCC T-3′), targeting FOXP3 or a non-target shRNA control in PGLV3-H1 vector (Shanghai, China). Target cells were infected with filtered lenti-virus plus 6 μg/mL polybrene (Sigma-Aldrich) to generate stable cell lines [20].

### Isolation of CD4^+^CD25^+^ T cells and CD4^−^CD25^−^ T cells

Heparin-treated blood was obtained from 3 donors and peripheral blood mononuclear cells (PBMC) PBMC were isolated by centrifugation over lymphocyte separation medium (Sigma-Aldrich). CD4^+^CD25^+^ T cells and CD4^−^CD25^−^ T cells which were sorted by magnetic beads (Miltenyi Biotec) from PBMC were used as positive and negative controls and cultured in RPMI-1640 (Invitrogen) at 37 °C in a humidified atmosphere containing 5% CO_2_.

### Laser capture microdissection

Immunohistochemical staining was performed on frozen sections of 15 HCC patients who underwent primary and curative resection in Liver Cancer Institute, Zhongshan Hospital of Fudan University to identify and isolate tumor cells from other immunocytes, allowing a more precise microdissection. Microdissection was performed by a PixCell laser capture microscope with an infrared diode laser (Arcturus Engineering, Santa Clara). Primary HCC cells were captured for RNA extraction by focal melting of the membrane through laser activation.

### Real-time RT-PCR analysis

Real-time RT-PCR was performed as described in Additional file 1: Supplementary Methods.

### Western blotting analysis

Western blotting was performed as described previously [21] with recommended concentrations of antibodies. More details were in Additional file 1: Supplementary Methods.

### Immunofluorescence

The expression of FOXP3 in Hep3B and 97H cells was detected by immunofluorescence, which was performed as previously described [22], using mouse anti-human FOXP3 Ab (1:200 dilution, Santa Cruz Biotechnology) and a goat anti-mouse IgG-FITC antibody (Invitrogen). Images were acquired using a LSM510 Confocal Laser Scanning Microscope (Carl Zeiss).

### Tissue samples

Paraffin-embedded tissue samples were selected from 240 HCC patients who underwent primary and curative resection for their tumor in Liver Cancer Institute, Zhongshan Hospital of Fudan University (Shanghai, China) between 2002 and 2006, as previously described [23]. Follow-up procedures and post-operative treatments according to a uniform guideline were described previously [24]. Tumor differentiation was graded using the Edmondson grading system. Clinical staging was according to the 7th edition of AJCC/UICC TNM classification system. Conventional clinicopathologic variables are detailed in Table 1. Overall survival (OS) and time to recurrence (TTR) were calculated from the date of surgery to the date of the first recurrence and death, respectively. Data were censored at the last follow-up for patients without relapse, or death. The median follow-up period was 39.0 months (range, 1.5–95.0; SD, 22.7).

**Table 1:** Correlation between FOXP3 expressin in tumor cells and clinicopathologic characteristics (*n* = 240)

Variables	FOXP3 staining		*P*
	Low (*n* = 125)	High (*n* = 115)	
	*n* (%)	*n* (%)	
Age (years)			
≤ 50	59 (47.2)	53 (46.1)	0.897
> 50	66 (52.8)	62 (53.9)	
Gender			
Male	103 (82.4)	101 (87.8)	0.280
Female	22 (17.6)	14 (12.2)	
HBsAg			
Negative	10 (8.0)	7 (6.1)	0.622
Positive	115 (92.0)	108 (93.9)	
Liver cirrhosis			
No	12 (9.6)	16 (13.9)	0.321
Yes	113 (90.4)	99 (86.1)	
Serum AFP (ng/mL)			
≤ 20	34 (27.2)	48 (41.7)	0.021
> 20	91 (72.8)	67 (58.3)	
Tumor size (cm)			
≤ 5	57 (45.6)	60 (52.2)	0.366
> 5	68 (54.4)	55 (47.8)	
Tumor number			
Single	98 (78.4)	86 (74.8)	0.543
Multiple	27 (21.6)	29 (25.2)	
Tumor encapsulation			
Yes	58 (46.4)	61 (53.0)	0.366
No	67 (53.6)	54 (47.0)	
Vascular invasion			
No	53 (42.4)	72 (62.6)	0.002
Yes	72 (57.6)	43 (37.4)	
Tumor differentiation			
I/II	71 (56.8)	64 (55.7)	0.897
III/IV	54 (43.2)	51 (44.3)	
BCLC			
0/A	54 (43.2)	60 (52.2)	0.196
B/C	71 (56.8)	55 (47.8)	
TNM stage			
I	46 (36.8)	60 (52.2)	0.019
II/III	79 (63.2)	55 (47.8)	

### Tissue microarray construction and immunohistochemistry

Tissue microarrays (TMAs) were generated as previously described [21], and immunohistochemistry for FOXP3 was carried out on TMAs as previously described [12]. More details were in Additional file 1: Supplementary Methods.

### Cell proliferation and migration assays

Cell proliferation and migration assays were described in the Supplementary Methods. All tests and analyses were carried out in triplicate.

### Xenograft tumor models

We performed subcutaneous tumorigenicity assays in BALB/c nude mice as described in Additional file 1: Supplementary Methods.

### Chromatin immunoprecipitation (ChIP)

For FOXP3 ChIP, goat polyclonal Ab to recombinant Foxp3 protein (ChIP Grade, Abcam) was used. ‘Flow-through’ IgG devoid of FOXP3 antibodies served as a negative control. Antibodies specific for different histone modifications were from Upstate Biotechnology. ChIP was carried out as described elsewhere [25]. Relative abundance of regions of interest in precipitated DNA was measured by qPCR using Power SYBR Green PCR master mix (Applied Biosystems).

### Statistical analysis

All statistical analyses were performed with SPSS version 16.0 software. The data were expressed as mean ± standard deviation (SD). The association between variables was analyzed using Student’s *t*-test, Mann–Whitney *U* test or Fisher’s exact test, as appropriate. Survival curves were obtained by the Kaplan–Meier method using a log-rank test. Cox regression analysis was used to evaluate the prognostic significance. *P*-values <0.05 were considered to be statistically significant.

## Results

### FOXP3 Expression in HCC cell lines and tissues

**Fig. 1:**
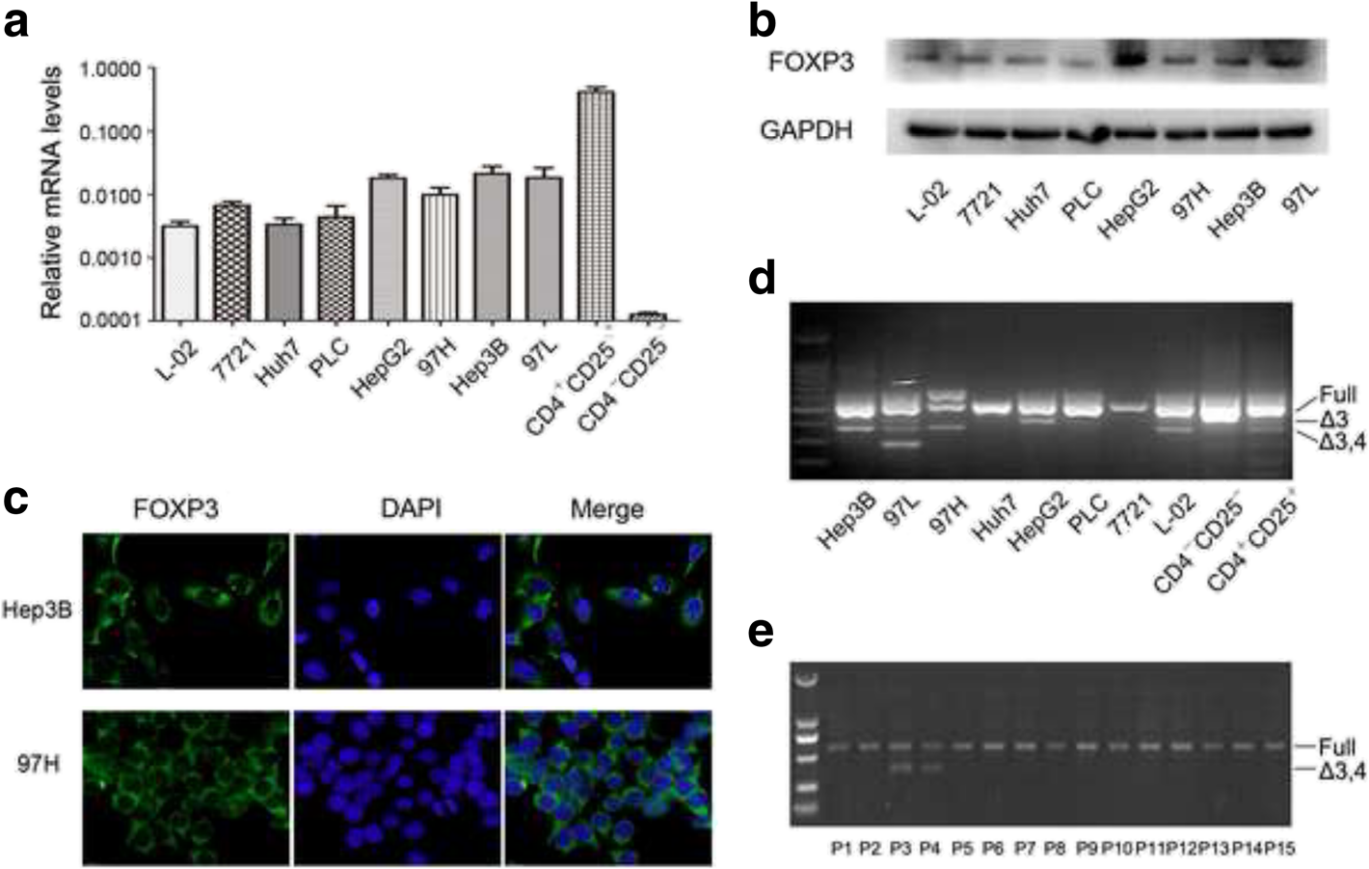
Expression profile of FOXP3 in human HCC. **a** Real-time RT-PCR analysis of FOXP3 expression in a normal liver cell line and HCC cell lines, using T cells as control. **b** Western blotting analysis of the protein levels of FOXP3 in a normal liver cell line and HCC cell lines. **c** Immunofluorescence images of FOXP3 expression Hep3B and 97H cells. **d** FOXP3 splice variants were detected in HCC cell lines by PCR analysis. Full, full-length; Δ3, the splice variant with exons 3 deleted; Δ3,4, the splice variant with exons 3 and 4 deleted. **e** FOXP3 splice variant with exons 3 and 4 deleted was found in 2/15 HCC patients

FOXP3 mRNA expression was clearly detectable in all HCC cell lines and a normal liver cell line L-02, using CD4^+^CD25^+^ T cells as a positive control and CD4^+^CD25^−^ T cells as the negative control (Fig. 1a).

Then, Western blotting revealed obvious FOXP3 protein expression in HCC cell lines and normal liver cell L-02 (Fig. 1b). Notably, the immunofluorescence assays proved the expression of FOXP3 in Hep3B and 97H cells as examples and FOXP3 staining was localized predominantly in the cytoplasm (Fig. 1c).

The FOXP3 transcripts in HCC cell lines were amplified using primers spanning exons 1–12, and then the PCR products were sequenced. As shown in Fig. 1d, Huh7, PLC and 7721 only expressed the full-length FOXP3, while other tumor cell lines also expressed the splice variants as observed in the T cells. In addition, three tumor cell lines (Hep3B, 97 L and 97H) as well as L-02 cells expressed a splice variant with both exons 3 and 4 deleted, similar to but slightly different to that identified in breast cancers and melanoma [10, 26]. The alternative deletion mutation resulted in a frame shift beginning with codon 70 and a premature termination at codon 151. Importantly, the splice variant lacking both exons 3 and 4 were detected in 13.3% (2/15 cases) of laser captured microdissection of primary HCC cells (Fig. 1e).

### Evaluation of FOXP3 expression as a prognostic factor in human HCC

We next evaluated the value of tumor cell FOXP3 expression in predicting clinical outcome in a cohort of 240 HCC patients by immunohistochemistry. The relationship between FOXP3 expression and clinicopathological features showed that high expression of FOXP3 correlated significantly with low serum AFP level (*P* = 0.021), absence of vascular invasion (*P* = 0.002) and early TNM stage (*P* = 0.019), suggesting an tumor-inhibiting effect of FOXP3 in HCC (Table 1). In our cohort, only 16 patients have hepatitis C infection (6.7%), thus, we exclude the hepatitis C infection from clinicopathologic characteristics.

**Fig. 2:**
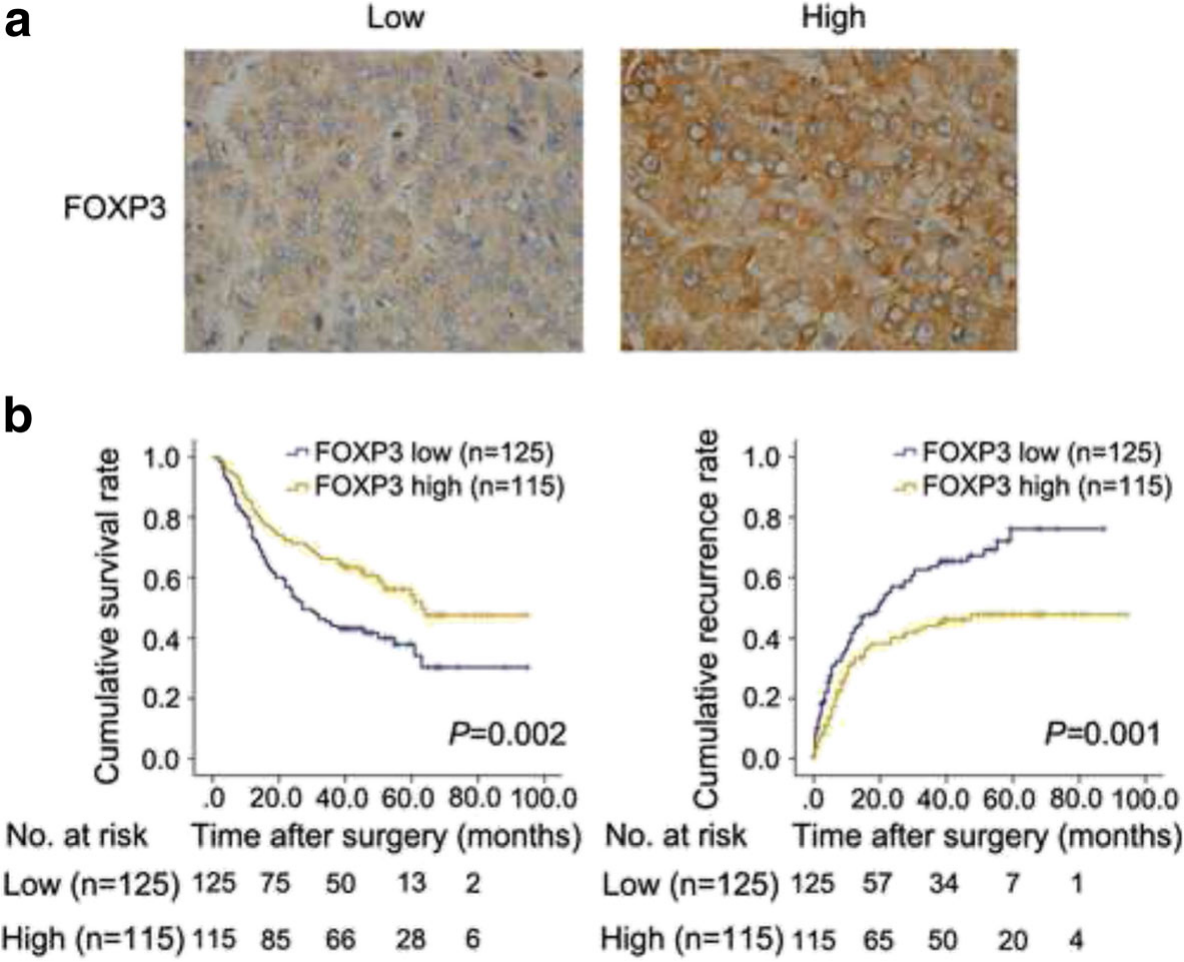
FOXP3 predicted a favorable prognosis in HCC patients. **a** The representative low expression and high expression of FOXP3 in HCC tissue. **b** The OS and TTR for the low and high FOXP3 expression groups were significantly different (by the 2-sided log-rank test) in the cohort of 240 patients. The absolute number of patients at risk is listed below each curve

In univariate analysis, conventional clinicopathological features which correlated with prolonged OS and TTR were low serum AFP level, small tumor size(*<*5 cm), single tumor, complete tumor capsule, absence of vascular invasion and early tumor stage, while the absence of liver cirrhosis and well differentiation were significantly associated just with better OS (Table 2). In particular, FOXP3-high patients showed significantly increased OS and TTR than FOXP3-low patients (Estimated mean OS 60.0 versus 44.8 months, *P* = 0.002; Estimated mean TTR 56.1 versus 34.9 months, *P* = 0.001, respectively; Fig. 2a and b).

**Table 2:** Univariate and multivariate analyses of factors associated with OS and TTR (n = 240)

Variables	OS			TTR		
		Multivariate			Multivariate	
	Univariate *P*	HR (95.0% CI)	*P*	Univariate *P*	HR (95.0% CI)	*P*
Age, >50 vs. ≤50 years	0.169	NA		0.289	NA	
Gender, Male vs. Female	0.259	NA		0.084	NA	
HBsAg, Positive vs. negative	0.264	NA		0.171	NA	
Liver cirrhosis, Yes vs. No	0.019	1.77 (0.89–3.52)	0.104	0.150	NA	
Serum AFP, >20 vs. ≤20 ng/mL	<0.001	1.23 (0.80–1.88)	0.350	0.002	1.31 (0.88–1.94)	0.181
Tumor size, >5 vs. ≤ 5 cm	<0.001	1.16 (0.56–2.43)	0.692	<0.001	1.57 (0.75–3.26)	0.231
Tumor number, Multiple vs. Single	0.001	1.78 (1.13–2.80)	0.013	<0.001	2.34 (1.48–3.70)	<0.001
Tumor encapsulation, No vs. Yes	0.011	1.06 (0.73–1.53)	0.770	<0.001	1.54 (1.07–2.22)	0.021
Vascular invasion, Yes vs. No	<0.001	4.42 (2.26–8.63)	<0.001	<0.001	4.28 (2.30–7.97)	<0.001
Tumor differentiation, III/IV vs. I/II	0.002	1.74 (1.20–2.53)	0.004	0.077	NA	
TNM stage, II /III vs. I	<0.001	0.72 (0.35–1.51)	0.389	<0.001	0.73 (0.37–1.45)	0.368
BCLC stage, B/C vs. 0/A	<0.001	1.30 (0.59–2.86)	0.521	<0.001	0.76 (0.35–1.64)	0.478
FOXP3, High vs. Low	0.003	0.64 (0.44–0.92)	0.017	0.001	0.66 (0.46–0.95)	0.027

Multivariate Cox regression analysis was performed using significant factors (*P <* 0.05) in univariate analysis along with FOXP3 expression status. The results proved FOXP3 as an independent predictor of both OS [hazard ratio (HR) = 0.64; 95% confidence interval (CI) = 0.44–0.92; *P* = 0.017] and TTR (HR = 0.66, 95% CI = 0.46–0.95; *P* = 0.027; Table 2). Survival analysis was clearly indicated that the expression of FOXP3 was a significant and independent predictor of HCC patient outcome.

### FOXP3 Reduces proliferation, migration and invasion of HCC cells in vitro, as well as tumor growth in vivo

**Fig. 3:**
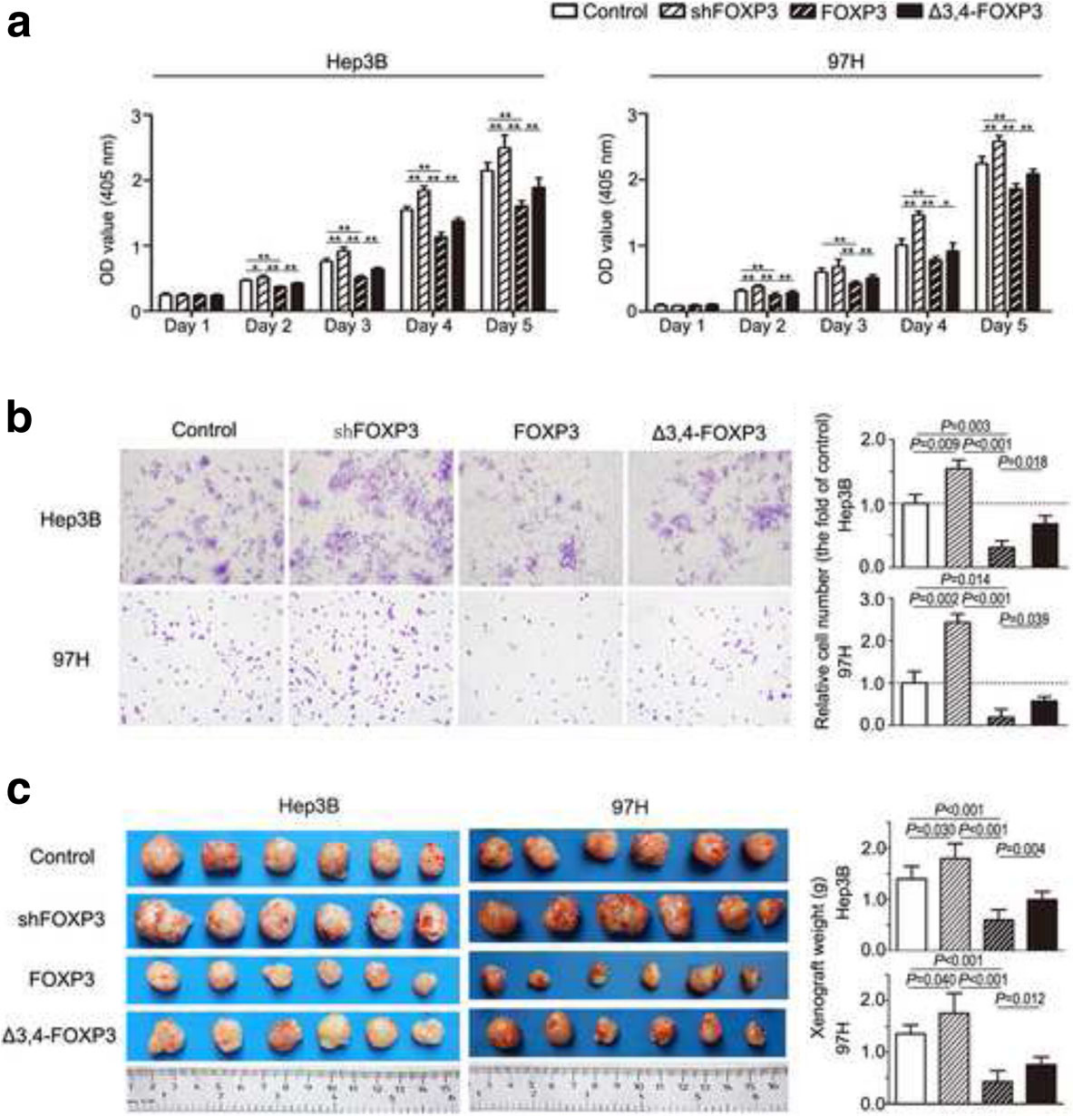
FOXP3 and FOXP3 splice variant inhibited HCC proliferation and invasion. **a** Proliferation assays were visualized as histogram. *P < 0.05, **P < 0.01; Δ3,4-FOXP3, the FOXP3 splice variant with exons 3 and 4 deleted. **b** The invasion of cancer cells was measured by matrigel-coated transwell assay. **c** Morphologic characteristics of subcutaneous tumor xenografts. Significant differences in tumor weight were revealed between the experimental and control groups (*n* = 6)

To substantiate tumor suppressive activity of FOXP3, we knocked down and over-expressed FOXP3 in HCC cell lines Hep3B (Hep3B-shFOXP3 and Hep3B-FOXP3, respectively) and 97H (97H–shFOXP3 and 97H–FOXP3, respectively). Previous studies have indicated that artificially designed deletions of the N-terminal portion, C-terminal portion, zinc finger domain or leucine zipper domain of FOXP3 resulted in reduced tumor suppressive activities. However, the exact significance and biological relevance of the naturally detected Δ3,4-FOXP3 remains elusive. We thus cloned and transfected Δ3,4-FOXP3 cDNA into HCC cell lines Hep3B and 97H (Hep3B-Δ3,4-FOXP3 and 97H-Δ3,4-FOXP3, respectively). We found that knock-down of FOXP3 in Hep3B and 97H cells led to obviously enhanced tumor proliferation (*P < 0.05, **P < 0.01; Fig. 3a) and cell migration (*P* = 0.009 and *P* = 0.002 for Hep3B and 97H, respectively; Fig. 3b) compared to control cells in vitro. In contrast, over-expression of FOXP3 in Hep3B and 97H cells resulted in significantly reduced tumor proliferation (*P < 0.05, **P < 0.01; Fig. 3a) and cell migration (*P* = 0.003 and *P* = 0.014 for Hep3B and 97H, respectively; Fig. 3b) compared to control cells. Additionally, over-expression of Δ3,4-FOXP3 showed a significantly reduced inhibition on tumor growth (*P < 0.05, **P < 0.01; Fig. 3a) and migration (*P* = 0.018 and *P* = 0.039 for Hep3B and 97H, respectively; Fig. 3b) than full-length FOXP3. The results further indicated that FOXP3 played a critical role in suppressing HCC growth and invasion.

To evaluate the impact of FOXP3 on tumorigenicity in vivo, transfected tumor cell lines and their respective controls were subcutaneously injected into nude mice. The tumor cells with down-regulation of FOXP3 grew to a significantly heavier weight (*P* = 0.030 and *P* = 0.040 for Hep3B and 97H, respectively; Fig. 3c) compared with controls. Meanwhile, up-regulation of FOXP3 in Hep3B and 97H inhibited their subcutaneous tumorigenesis (*P* < 0.001 and *P* < 0.001 for Hep3B and 97H, respectively; Fig. 3c). However, over-expression of Δ3,4-FOXP3 showed a remarkable loss of tumor suppression than full-length FOXP3 (*P* = 0.004 and *P* = 0.012 for Hep3B and 97H, respectively; Fig. 3c).

### FOXP3 Targeted genes in HCC

**Fig. 4:**
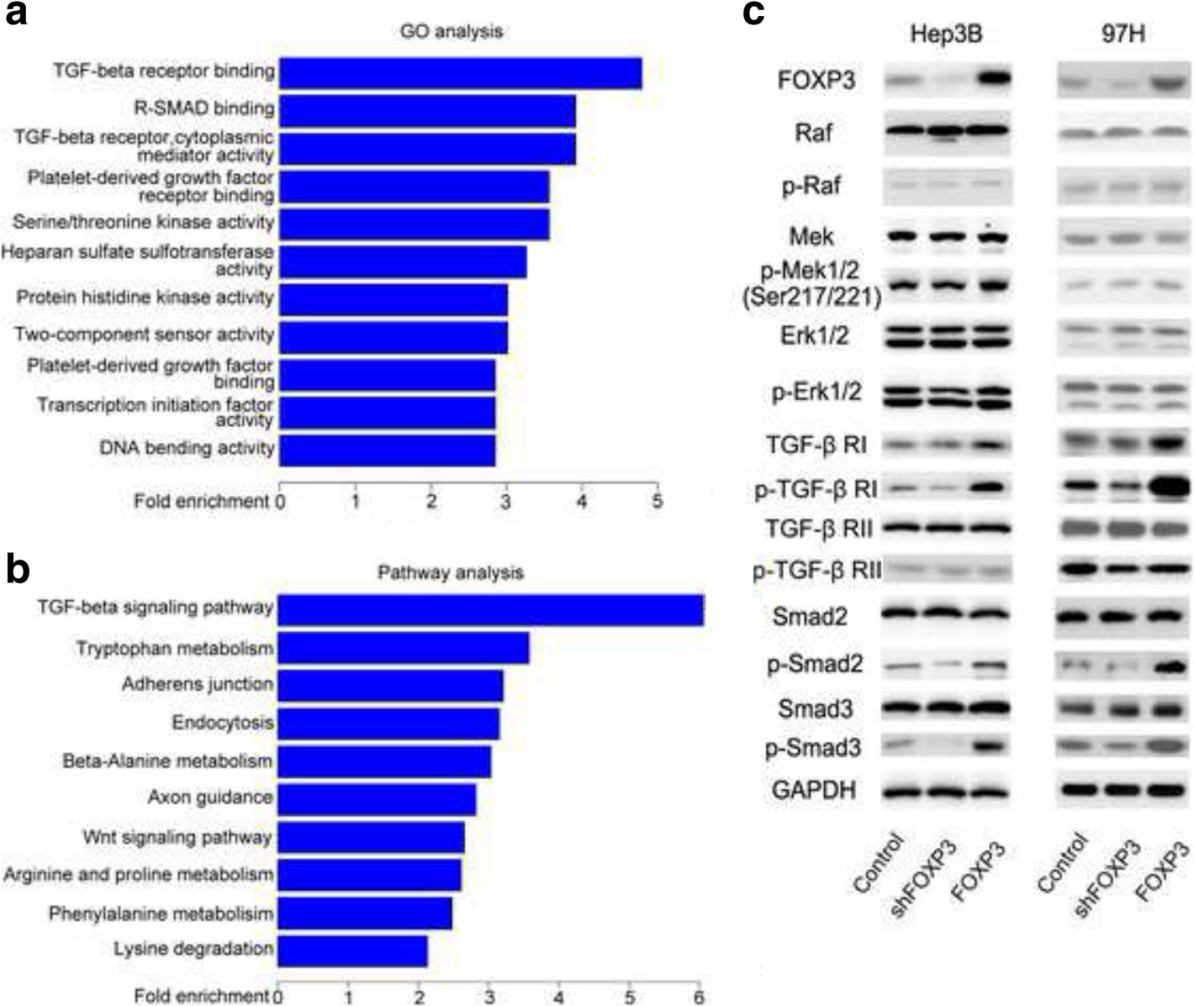
FOXP3 inhibited HCC progression by enhancing TGF-β signaling pathway. **a** and **b** TGF-β signaling pathway was screened out by Gene ontology (GO) analysis and Pathway analysis as a candidate pathway which was correlated with FOXP3. **c** The expression and phosphorylation of indicated molecules were detected by Western blotting

Using ChIP sequencing, we evaluated FOXP3 DNA binding in pcDNA-FOXP3 Hep3B and 97H, from immunoprecipitated DNA fragments. The DNA precipitated by control-IgG was used as a control. Given that most of the FOXP3-binding sites reside within 2 kb of TSS [27], we defined direct target genes with FOXP3 binding within 2 kb of their TSS in both Hep3B and 97H cells and identified a common gene pool of 2853 genes. Importantly, Gene ontology (GO) analysis indicated that FOXP3 may exert tumor inhibition effects by affecting TGF-β receptor binding activity, and pathway analysis showed that FOXP3-induced gene regulation may be controlled by TGF-β signaling pathway (Fig. 4a and b). To gain insight into the mechanism by which FOXP3 inhibited HCC growth and invasion, a panel of signaling pathways that may relate to HCC progression and especially TGF-β pathway were examined by western blotting. The phosphorylation of TGF-β RI was reduced when FOXP3 was knocked down, and enhanced remarkably as FOXP3 was over-expressed (Fig. 4c). Immunoblotting further revealed that FOXP3 deficiency decreased the phosphorylation of Smad2/3, and over-expression of FOXP3 triggered the phosphorylation of Smad2/3 (Fig. 4c).

In conclusion, our studies suggested that FOXP3 inhibited HCC progression by enhancing the phosphorylation of TGF-β RI and activating TGF-β/Smad2/3 signaling pathway.

## Discussion

FOXP3 is a member of the forkhead/winged-helix gene family of transcription factors associated with activation and differentiation of Treg cells [28]. Recently, the expression of FOXP3 has been recently reported in non-hematopoietic derived cells, including normal epithelial cells and some tumor cells from different tissue origins [29]. In this study, the expression of FOXP3 was detected in HCC cell lines and tumor tissues, although the expression was obviously lower than CD4^+^CD25^+^ cells. Immunostaining of FOXP3 in Hep3B and 97H cells indicated that FOXP3 staining was localized predominantly in the cytoplasm. The cytoplasmic localization of FOXP3 was detected in several human cancers, including human breast carcinoma [30]. Foxp3 molecule includes a winged-helix/forkhead (FKH) domain that carries a supposed nuclear localization signal. Mutations of this domain were observed in the Scurfy mouse and in the corresponding human IPEX syndrome, leading to a cytoplasmic accumulation of FOXP3 [31].

Previous data derived from several in vitro studies increasingly pointed to the critical role of FOXP3 as a tumor suppressor, including repressing tumor cell proliferation, promoting apoptosis and reducing cell invasion in at least breast, prostate and ovarian carcinoma models [10, 12, 32]. However, there were conflicting data suggesting a strong correlation of elevated FOXP3 expression in cancer cells with poor survival and appearance of lymph node metastases in gastric tumor, urinary bladder cancer and esophageal squamous carcinoma [33–35]. The reason for this discrepancy remains unclear, but Triulzi et al. considered it might be explained by a loss of oncosuppressive function of FOXP3 in human tumors due to breaking-function mutations [5]. Our tissue microarray data showed that high expression of FOXP3 in tumor tissue significantly correlated with low serum AFP level, absence of vascular invasion and early TNM stage, suggesting a negative regulatory role of FOXP3 in HCC progression. Although over half of the patients in advanced HCC with BCLC B/C stadium expressed low FOXP3, the inverse correlation of FOXP3 and BCLC stadium was insignificant (*P* = 0.196) probably due to small sample size (Table 1). Coincidently, survival analysis revealed that patient with high level of FOXP3 in tumor cells was remarkably and independently associated with improved survival and reduced recurrence, regarding FOXP3 as an independent prognostic factor of HCC patient outcome. Furthermore, over-expression of FOXP3 obviously inhibited tumor proliferation and invasion, while FOXP3 down-regulation resulted in enhanced tumor growth and invasion. Additionally, we detected the deletion mutation of FOXP3 in HCC cell lines and tissue samples. With the data of our cell and animal models, we found that Δ3,4-FOXP3 showed a moderate inhibition on tumor growth and migration than full-length FOXP3, indicating a restricted tumor-suppressive function probably caused by exon loss. Previously, FOXP3 was identified as a key player in mediating Treg inhibitory functions. Hence, our study provided additional information on the anti-tumor effect of FOXP3 in human HCC and was the first to demonstrate FOXP3 as a putative tumor suppressor in HCC progression.

Although the mechanism underlying the tumor suppression function of FOXP3 is not fully characterized, several genes and pathways have been found to be closely related. FOXP3 was found to inhibit tumor cell growth, serving as an important repressor for breast cancer oncogene SKP2 and HER2 [10, 11], and FOXP3–miR-146–NF-kB Axis has been suggested in leading to apoptosis during tumor initiation and tumor suppression in prostate cancer [36]. In our study, TGF-β signaling pathway was elucidated to correlate with FOXP3 by pathway analysis, and furthermore the FOXP3-related regulation of TGF-β/Smad2/3 was confirmed by western blotting assays. Smad2 and Smad3 are receptor-associated R-Smads which form the classical TGF-β signaling cascade [37]. It is widely accepted that TGF-β plays a bifunctional roles in tumor suppression and tumor promotion [38]. In a Japanese study, TGF-β acted as a tumor suppressor by transmitting a signal through pSmad3C (phosphorylate Smad3 at the COOH-terminal) and participating in the cytostatic response by repressing *c-Myc* gene, and on the other hand, pSmad2/3 L (phosphorylate Smad2 and Smad3 at the linker regions) signaling had oncogenic potential on tumor growth and invasion via up-regulation of c-Myc and MMP [39]. Their results indicated the reversibility of Smad-dependent signaling between tumor suppression and oncogenesis. Interestingly, a recent study showed that TGF-β could drive tumor suppression in pancreatic cancer cells which underwent a lethal epithelial-mesenchymal transition (EMT) by converting TGF-β-induced Sox4 from an enforcer of tumorigenesis into a promoter of apoptosis [40]. All these studies provided us novel ideas in searching for further understandings of tumor inhibition via FOXP3/TGF-β/Smad2/3 pathway.

## Conclusions

In summary, we demonstrated that FOXP3 was expressed in tumor cells and an increased expression of FOXP3 was associated with better survival and reduced recurrence. In mechanism, FOXP3 exerted its tumor inhibition effects probably via the regulation of TGF-β/Smad2/3 pathway. Thus, there is a strong tendency to believe FOXP3 as a potential tumor suppressor in HCC. Although this is a single-center study and the FOXP3’s exact role in cancer cells remains open-ended, the identification of molecules related to FOXP3 expression and function in tumor cells would provide additional information on understanding the biological behavior of HCC.

## Additional files

Additional file 1: Supplementary Methods. (DOC 37 kb)

Additional file 2: The additional file contains 6 sub-files. (ZIP 2185 kb)

## Abbreviations

AFP: α-fetoprotein
ChIP: Chromatin immunoprecipitation
CI: Confidence interval
EMT: Epithelial-mesenchymal transition
FOXP3: Forkhead box P3
HCC: Hepatocellular carcinoma
OS: Overall survival
PBMC: Peripheral blood mononuclear cell
SD: Standard deviation
TGF-β: Transforming growth factor-β
TMA: Tissue microarray
TTR: Time to recurrence
