# Supplementary material for: FOXP3 Is a HCC suppressor gene and Acts through regulating the TGF-β/Smad2/3 signaling pathway
Source: BMC Cancer. 2017 Sep 13;17:648. doi: 10.1186/s12885-017-3633-6 (PMC5598072; doi:10.1186/s12885-017-3633-6)
Supplement: Supplementary file 1 — Supplementary Methods. (DOC 37 kb) [file 12885_2017_3633_MOESM1_ESM.doc]

**Supplementary Methods**

**Real-time RT-PCR**

Total RNA isolation, purification and reverse transcription were conducted as previously described . Real-time PCR was performed using a SYBR-Green PCR master mix (Invitrogen) and ABI PRISM 7500 Sequence Detection System (Applied Biosystems), according to the manufacturers’ instructions. All samples were performed in triplicate and ACTB values were used to normalize gene expression using 2^-ΔCT^ method.

**Western blotting analysis**

Total protein was prepared from the cell lines which lysed in RIPA buffer containing protease inhibitors. The primary antibody was mouse anti-human FOXP3 Ab (1:200 dilution, Santa Cruz Biotechnology). Horseradish peroxidase (HRP)-conjugated secondary antibodies and enhanced chemiluminescence (ECL) reagents were from Amersham. GAPDH (1:2000 dilution, Sigma-Aldrich) was used as the loading control. Rabbit monoclonal antibodies for signal molecules were purchased from Cell Signaling Technology.

**Tissue microarray construction and immunohistochemistry**

Core samples were obtained from representative regions from each tumor on hematoxylin and eosin staining. Triplicate 1-mm cores were taken from different areas of the same tissue block for each case (tumor tissue and matched noncancerous liver tissue). Tissue microarrays were constructed using an arraying machine (Beecher Instruments).

For immunostaining, 4-μm sections were deparaffinized and subjected to antigen retrieval (citrate buffer, pH=6.0). Sections were then incubated for 30 min with goat polyclonal antibody to FOXP3 (1:100 dilution; Santa Cruz Biotechnology). Reaction products were visualized with 3, 3’-diaminobenzidine tetrahydrochloride and counterstained with hematoxylin. FOXP3 immunostaining intensity was dichotomised (low and high) by two independent investigators blinded to the clinicopathologic data.

**Cell proliferation and migration assays**

Cell proliferation assay was determined by Cell-Counting Kit (CCK)-8 (Dojindo), reading absorbance at 450 nm according to the manufacturer’s instructions and as previously described . HCC cells were seeded at 1000 cells/well in 96-well plates.

*In vitro* migration assay was performed in Transwell Permeable Supports (Corning) with an 8-µm polycarbonate filter membrane essentially as described. 2×10^4^ cells were placed into the top chamber of each insert. The migrated cells were fixed and stained in dye solution containing 0.1% crystal violet and 20% methanol after 24 hours of incubation at 37°C. Cell migration was quantitated by visual inspection of the filter membranes. All results are representative of three independent experiments performed in triplicate.

**Subcutaneous tumor models**

Mice were manipulated and housed according to the protocols approved by the Shanghai Medical Experimental Animal Care Commission. To evaluated in vivo tumor growth, 5×10^6^ tumor cells were suspended in 100 μl serum-free DMEM and subcutaneously injected in the right ﬂank of each mouse respectively (6 in each group, male BALB/c-nu/nu, 6-8 weeks) as described previously. On day 28, mice were sacrificed, and tumors were photographed and weighed after being dissected.

**References**

[1] Gao Q, Zhao YJ, Wang XY, Qiu SJ, Shi YH, Sun J, et al. CXCR6 upregulation contributes to a proinflammatory tumor microenvironment that drives metastasis and poor patient outcomes in hepatocellular carcinoma. Cancer Res. 2012; 72: 3546-56.

[2] Jia D, Wei L, Guo W, Zha R, Bao M, Chen Z, et al. Genome-wide copy number analyses identified novel cancer genes in hepatocellular carcinoma. Hepatology. 2011; 54: 1227-36.
